# Autonomic responses during Gambling: the Effect of Outcome Type and Sex in a large community sample of young adults

**DOI:** 10.1007/s10899-022-10118-6

**Published:** 2022-04-09

**Authors:** Cathrine Hultman, Sofia Vadlin, Mattias Rehn, Guillaume Sescousse, Kent W Nilsson, Cecilia Åslund

**Affiliations:** 1 Centre for Clinical Research, Region Västmanland, Västmanland Hospital Västerås, Uppsala University, Västerås, Sweden; 2 Department of Public Health and Caring Sciences, Uppsala University, Uppsala, Sweden; 3 Lyon Neuroscience Research Center, PSYR2 Team, INSERM U1028—CNRS UMR5292, University of Lyon, Lyon, France; 4 Department of Neuroscience, Uppsala University, Uppsala, Sweden

**Keywords:** Gambling, Near-miss, Autonomic responses, Sex differences, Skin conductance, Heart rate

## Abstract

Psychological theories consider autonomic arousal to be a reinforcer for problem gambling. Structural characteristics such as near-misses, which are non-win events that come close to a real win, have been shown to elicit win-like responses while increasing motivation and gambling persistence. This study investigated the autonomic and subjective responses of young adults to different gambling outcomes. This study also investigated sex differences in autonomic and subjective responses to different gambling outcomes.

Participants from Sweden (n = 270) performed a computerized slot machine task that produced wins, near-misses (before and after payline) and full-misses. Phasic measurements of heart rate (HR) and skin conductance responses (SCR) were recorded during gambling performance and ratings of perceived chance of winning, pleasure and motivation to play were collected following each gambling outcome.

Autonomic responses differed across slot machine outcomes as indicated by HR and SCR. Compared with other gambling outcomes, near-misses elicited the largest HR accelerations, and they also elicited larger HR decelerations and SCRs relative to full-misses. Near-misses before and after payline elicited differential psychophysiological responses and subjective reports, suggesting different emotional processing of near-miss subtypes. Females showed increased SCRs and motivation following win outcomes compared with males.

In conclusion, wins, near-misses and full-misses generate differential physiological and subjective responses among young adults. Autonomic responses to wins differed between male and female players, emphasizing the need to consider sex differences when investigating the role of autonomic arousal in gambling.

## Introduction

Gambling is a recreational activity that can lead to problematic gambling behaviour associated with considerable harm. Gambling disorder was previously categorized as an impulse control disorder, but with the fifth edition of the *Diagnostic and Statistical Manual of Mental Disorders* (American Psychiatric Association, 2013), it was reclassified and grouped with substance-related and addictive disorders. Problem gambling is a subclinical term used in population surveys to estimate problematic gambling behaviour that precedes gambling disorder. Current estimates of problem gambling worldwide range between 0.12% and 5.8% (Calado & Griffiths, 2016).

Several psychological theories consider autonomic arousal to be important in the development and facilitation of problematic gambling behaviour (Blaszczynski & Nower, 2002; Brown, 1986; Sharpe, 2002; Sharpe & Tarrier, 1993). Previous psychophysiological research on gambling has been dominated by the use of tonic measures to assess general arousal, with levels averaged across long time periods or entire gambling sessions. Consistent results indicate increased arousal in problem gamblers relative to non-gamblers during gambling (Krueger et al., 2005; Ladouceur et al., 2003; Meyer et al., 2000, 2004). Tonic measures of autonomic arousal also capture reliable increases in skin conductance levels and heart rate (HR) following winning events (Coventry & Constable, 1999; Coventry & Hudson, 2001; Diskin & Hodgins, 2003; Moodie & Finnigan, 2005; Sharpe, 2004; Wilkes et al., 2010; Wulfert et al., 2005, 2008).

Although relatively scarce, there is an emerging body of research using phasic measures to associate distinct physiological responses to specific gambling outcomes. HR and skin conductance responses (SCRs) have proved to be reliable indexes of event-related responses to winning and losing outcomes in gambling (Dixon et al., 2010; Lole et al., 2012, 2014). While both HR and SCR can reliably capture autonomic changes, they reflect different psychological processes. Cardio-vascular patterns can be assessed relative to baseline HR to capture a biphasic cardio-vascular response, with an initial deceleration followed by subsequent acceleration in HR. Increased SCR and HR acceleration both reflect increased arousal as a preparatory response to appetitive or defensive stimuli (Bradley et al., 2001), but HR can also be sensitive to emotional valence, whereas SCR is primarily an index of arousal (Bradley et al., 2001; Codispoti et al., 2001; Lang et al., 1993). Large cardiac deceleration following sensory input is thought to be indicative of enhanced interest and orienting (Bradley et al., 2001; Codispoti et al., 2001). For example, when people view emotional pictures, unpleasant stimuli initially elicit a large cardiac deceleration.

### Near-miss Outcomes in Gambling

Another important issue in gambling research is the effect of structural characteristics in games that may be responsible for the reinforcement and facilitation of excessive gambling (Griffiths, 1993). One of the structural characteristics believed to be associated with the misuse potential of gambling games is near-misses (losing outcomes that come close to but fall just short of a successful outcome) (Reid, 1986).

Early psychological theories regarding near-misses emphasized their potential to generate win-like experiences and increase the sense of being close to a real win despite being objectively equal to a full-miss (Griffiths, 1993; Parke & Griffiths, 2006; Reid, 1986). Previous research found that moderate rates of near-miss outcomes in slot machines (around 30%) lead to prolonged gambling (Côté et al., 2003; Kassinove & Schare, 2001; MacLin et al., 2007). Studies have also shown that even though near-misses are generally experienced as negatively valenced and frustrating, they simultaneously increase the motivation to continue playing (Billieux et al., 2012; Clark et al., 2009, 2012, 2013; Qi et al., 2011; Stange et al., 2016, 2017).

Research on near-misses generally reports significant increases in SCRs following near-miss gambling outcomes, but with inconsistent results regarding the size of such increases relative to those that follow wins and full-misses. Some studies report larger SCRs following near-misses relative to all other outcomes, including wins (Dixon et al., 2011). However, most studies report larger SCRs for near-misses than for full-misses, but not larger than for wins (Clark et al., 2012, 2013; Sharman et al., 2015). One study failed to observe a difference in SCRs between near-misses and full-misses (Lole et al., 2012). Increased SCRs to near-misses have also been related to more severe gambling problems (Ulrich et al., 2016).

The patterns of cardio-vascular responses produced by near-misses also vary. One study found that changes in heart period for near-misses were comparable with those for wins (Ulrich et al., 2016.) Clark et al., (2012) found large HR acceleration to be most prominent for near-misses compared with other outcomes, whereas Dixon et al., (2011) observed larger HR deceleration to near-misses relative to other outcomes. Using the same approach, Clark et al., (2013) and Lole et al., (2012) failed to find a difference in HR between near-misses and full-misses.

In addition, brain imaging studies found that near-miss outcomes in a slot-machine gambling task recruited striatal and insula circuitry that are typically activated when experiencing a win, suggesting a win-like response to near-misses (Chase & Clark, 2010; Clark et al., 2009). The increased activation in these regions was especially prominent among problem gamblers (Chase & Clark, 2010; Dymond et al., 2014; Sescousse et al., 2016).

### Differential Effects of Near-miss Subtypes

Two types of near-misses defined by their position just before or after the winning position appear to generate differential self-ratings and physiological responses. Evidence from studies using a slot machine task indicates that near-misses in which the reel stops just before the payline are perceived as more pleasant and are associated with increased motivation to play. In contrast, near-misses in which the reel passes through the payline to stop on the next position are primarily perceived as aversive and demotivating (Clark et al., 2013; Sharman et al., 2015) also reported larger SCRs following near-misses that came after the payline compared with both near-misses that came before and regular full-misses.

The differential effects observed for these subtypes of near-misses may represent a distinctive effect caused by two directions of counterfactual processing, which is known to affect both emotion and motivation (Epstude & Roese, 2008; Markman & McMullen, 2003; Roese, 1994, 1997). Near-misses can be viewed as upward counterfactuals in which players mentally simulate a better outcome (“I almost hit the jackpot”). Near-misses in which the reel stops just before the winning position can be seen as additive upward counterfactuals that lead players to mentally simulate a trajectory towards a winning outcome as they anticipate a possible win, which increases their motivation to play. In contrast, near-misses in which the reel stops just after the winning position can trigger a process whereby the player mentally reverses the subsequent step, thus constituting a subtractive upward counterfactual, which is associated with a sense of frustration and regret (Clark et al., 2013; Markman & McMullen, 2003; Sharman et al., 2015; Wu et al., 2017). This hypothesis was further strengthened by Wu et al., (2017), who reported that players of a ‘wheel of fortune’ game felt unluckier and decreased their betting amounts following near-misses that occurred after the payline (subtractive counterfactual) compared with near-misses that occurred before the payline (additive counterfactual).

### Sex Differences

Gambling has traditionally been more prevalent among men than women. Consistent findings suggest that patterns of gambling behaviours in the general population differ between males and females, including gambling habits, gambling preferences, how and why gambling problems develop, and associated comorbidities. Women also tend to start gambling later in life than men, but they have a more rapid progression to gambling disorder, a phenomenon that has been termed telescoping. Furthermore, men tend to engage in more strategic forms of gambling, such as sports betting or poker, whereas women generally prefer non-strategic, chance-based online casino games, such as slot machines or bingo (Abbott et al., 2018; Grant et al., 2012; Hing et al., 2016; Håkansson & Widinghoff, 2020; Martins et al., 2002; Merkouris et al., 2016; Potenza et al., 2001, 2006; Stark et al., 2012; Sundqvist & Rosendahl, 2019; Tavares et al., 2003; Tschibelu & Elman, 2010; Zakiniaeiz et al., 2017).

However, whether these behavioural differences are accompanied by differences in autonomic responses to gambling outcomes is unclear. Franco et al., (2009) found greater increases in salivary cortisol levels among men compared with women while gambling on a horse race, suggesting sex differences in hypothalamic–pituitary–adrenal axis activation during gambling. One study used tonic measures of HR during fruit machine play to investigate sex differences in arousal during gambling and found no difference between men and women, albeit with a relatively small sample size (22 males, 20 females; (Coventry & Hudson, 2001). Wulfert et al., (2008) investigated the effects of expecting to win money on a horse race in two separate experiments, one with males and one with females. They found similar increases in HR for male and female players, but there were some differences in subjective excitement related to win size. Females showed the same level of excitement to small, medium and large wins, whereas males showed increased excitement to large compared with small and medium wins. However, this study did not statistically compare the males and females in terms of autonomic responses (Wulfert et al., 2008). Several studies that used neural responses observed sex differences in reward processing that were interpreted as greater emotional sensitivity to full-misses in women and greater sensitivity to reward in men (Dhingra et al., 2021; Garrido-Chaves et al., 2021; Grose-Fifer et al., 2014; Santesso et al., 2011).

### Objectives

The above findings show that research on autonomic arousal patterns associated with gambling characteristics, in terms of different outcomes, is still lacking in consistency. Apart from establishing the overall response patterns to different gambling outcomes, understanding the individual differences in affective processing during gambling is also crucial. Investigating the autonomic response patterns (via SCRs and HR) in relation to sex may offer important contributions to the psychophysiological research on gambling. Furthermore, improving reproducibility and the inclusion of large sample sizes are important concerns, and have received increased recognition especially within the field of psychology and neuroscience (Button et al., 2013; Stanley et al., 2018).

The aim of the current study was to investigate the phasic psychophysiological responses and subjective ratings (SRs) generated by win, near-miss and full-miss outcomes in a slot machine task, focusing on the differential effects of two subtypes of near-misses characterized by their position before or after the payline. A secondary aim was to investigate whether near-miss and full-miss outcomes are processed differently by male and female players with regard to physiological and subjective responses, using a large sample of young adults.

## Methods

### Participants

**Fig. 1:**
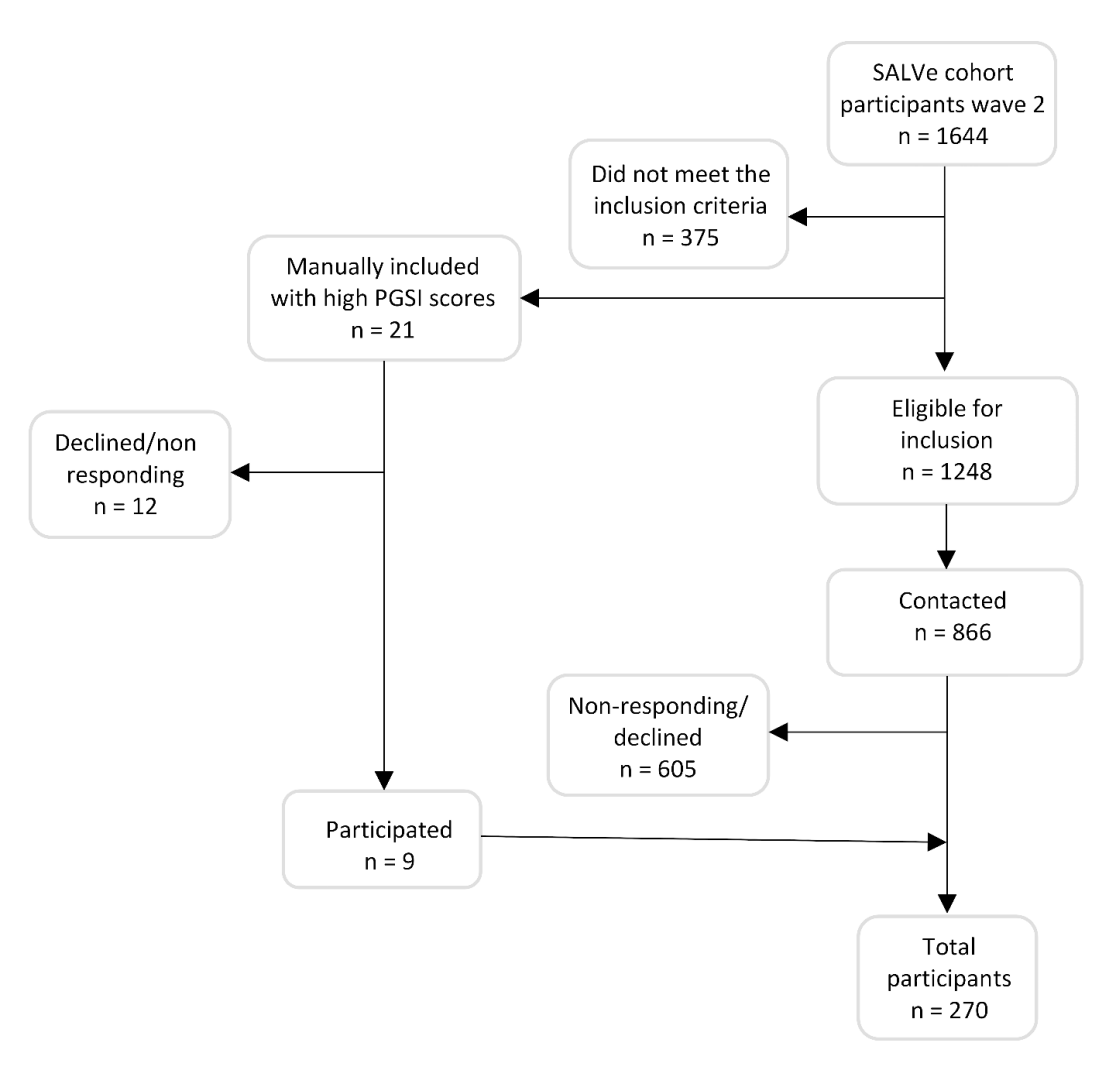
Inclusion procedure: total sample of participants

This study reports data from an extensive project that included several experimental tasks. Participants were drawn from a large cohort study of Swedish young adults born in 1997 and 1999 (Survey of Adolescent Life in Västmanland, SALVe Cohort). Participants were recruited from the cohort wave 2 (n = 1644) (for a full report on the cohort wave 2 inclusion, see Vadlin et al., (2018)). Cohort participants completed several questionnaires, including the Problem Gambling Severity Index (PGSI) (Ferris & Wynne, 2001), which is a 9-question screening tool that assesses problem gambling during the past 12 months. Scores on the PGSI range from 0 to 27, and participants with a score of ≥3 were included to maximize the number of gamblers in the study. To meet the inclusion criteria of the larger general project mentioned above (which addresses several objectives outside the scope of the current study), genetic information from the cohort participants was also required for eligibility. Eligible members of the cohort were contacted and willing participants were included in the study in a randomized order based on an even dispersion of age and sex until the final sample was reached ( n = 270, 140 women, 130 men, age 18–22 years; see Fig. 1).

Participants were invited to an experimental gambling session at the SALVe Science lab, Västmanland Hospital Västerås, Sweden. In the case of any previous history of gambling disorder participants would have been excluded from participation to avoid the risk of increasing the severity of gambling problems. Upon direct questioning, none of the participants reported a history of gambling disorder. There were no differences in socio-economic status (parents’ monthly income) or origin (parents born in/outside Scandinavia) between the participants in this study and the cohort (*p* = 0.690 and *p* = 0.893, respectively). All participants provided informed consent, and this study was approved by the Ethical Review Board of Uppsala (dnr 2016/569), with an extended approval (dnr: 2019 − 01368).

### Procedure

This study reports data from one of several tasks that participants performed during the same experimental session, including a battery of questionnaires on gambling, gaming, personality traits, sleep habits, sensory processing sensitivity, and positive/negative affect; four computerized cognition tasks; two interviews on substance and behavioural addictions; and three computerized gambling tasks. Psychophysiological responding was recorded throughout the session. Participants received a gift card of 1000 SEK (≈ 100 €) for participation in the entire session, with the possibility of additional 600 SEK (≈ 60 €, maximum of 200 SEK/gambling task ≈ 20 €) depending on their performance on the gambling tasks. The examiner provided detailed information about the procedures when informed consent was solicited, after which electrodes for recording HR and SCRs were attached and the first part of the session was administered (questionnaires, cognition tasks, and diagnostic interview on addictions). Participants had a short break with refreshments after the first part of the session and before starting the slot machine task. Electrodermal activity (EDA) and electrocardiograms (ECG) were recorded with a Biopac System MP150 (Biopac Systems, Goleta, CA, USA). The task computer was connected to the Biopac system and to a second computer running *Acqknowledge* v5.0.1 to event-mark the psychophysiological data using digital channels. For the skin conductance recording, two grounded Ag–AgCl electrodes were used (Biopac EL507 with a BN-PPGED amplifier module, sample rate 62.5, constant voltage 0.5 V, low-pass filter 3.0 Hz, high-pass filter DC) attached to the thenar and hypothenar eminences of the non-dominant hand. Electrode sites were cleaned using a dry cloth prior to the application. To facilitate recording of the EDA signal, 0.05 M NaCl electrolyte paste GEL101 was used. The signal was transformed into microsiemens (µS) in *Acqknowledge*. The ECG was recorded with three disposable grounded Ag–AgCl electrodes (Biopac EL504, with a BN-RSPEC module, sample rate 2000, low-pass filter 35 Hz, high-pass filter 1 Hz) attached to the right shoulder and grounded to the eighth rib on the left and right side. The electrodes contained liquid hydrogel (4% NaCl). The ECG data were converted into HR in *Acqknowledge*. Although room temperature and humidity were regulated, because of seasonal changes, the temperature ranged from 20 to 32 °C (M = 25.5 °C) and the humidity ranged from 20 to 58% (M = 41%) across the sessions.

### Slot Machine Gambling Task

**Fig. 2:**
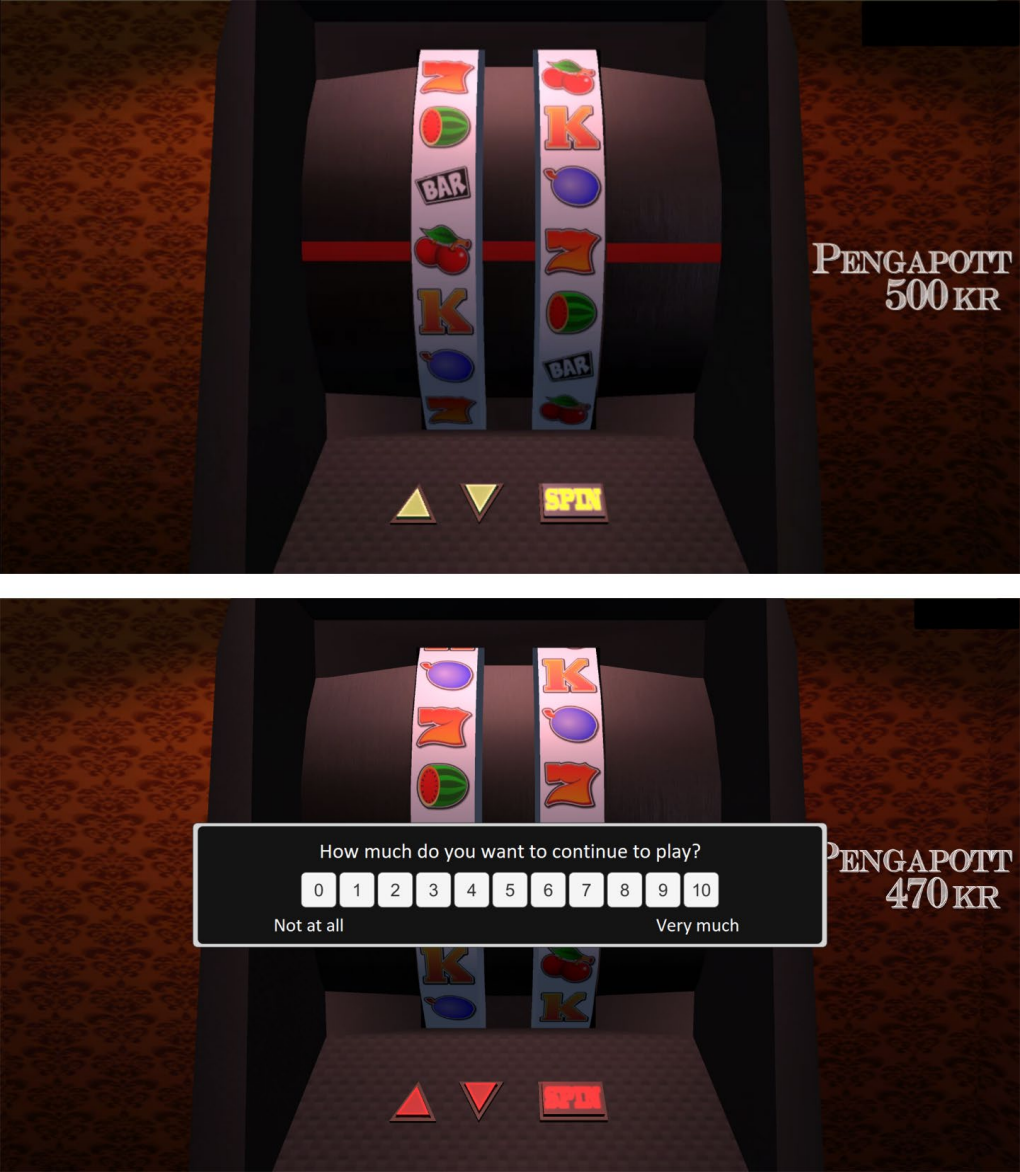
Screen display of the computerized slot machine task (reel display during selection/outcome and SRs)

The participants performed a computerized gambling task based on a slot machine gambling paradigm used in previous experiments by Clark et al., (2009; 2012; 2013) and Sescousse et al., (2016). The visual and auditory stimuli of the original task were modified to make the game more closely resemble a real slot machine. The slot machine task displayed two reels with six paired symbols and a payline placed horizontally across the centre of the reels (Fig. 2).

The participants performed three practice trials followed by the main session of 60 trials (approximate duration of 30 min). Each trial started with a selection phase during which participants scrolled the left reel to choose one of six icons, with no time constraints. They then spun the right reel, which decelerated to a standstill in 2.8–6.0 s (anticipation interval). A winning outcome occurred if the icons on the payline matched when the right reel stopped. Winning was followed by a short melody, applause and a message on the screen that read “Jackpot!” (5 s), followed by the message “You won 100 SEK” (4 s). There were two near-miss outcomes, one in which the matching icon stopped one position before the payline (‘near-miss before’) and one in which the matching icon stopped one position after the payline (‘near-miss after’). All other outcomes were defined as ‘full-misses’. The non-win outcomes were followed by a sound signifying a loss and a message on the screen that read “No win” (4 s). A running total of earnings was displayed on the right side of the screen throughout the task. Following each trial the participants made three SRs: “How pleased are you with the result?” (pleased with results), “How much do you want to continue to play?” (continue to play), and “How do you rate your chances of winning?” (chance of winning). Participants used the computer mouse to make their ratings for each question on an ordinal scale of 0–10 (with no time constraints).

Participants were given 500 SEK (≈ 50 €) to gamble with at the start of the slot machine task, with per trial wagers fixed at 15 SEK (≈ 1.5 €). The wager was deducted from their total following each losing outcome, and 100 SEK (≈ 10 €) was added to their total following each win. To increase their investment in the gambling task, the participants were told that they could receive up to 200 SEK (≈ 20 €) additional winnings depending on their overall success across the gambling trials. The task was standardized to produce the same distribution and order of outcomes for each session, which allowed for comparisons between individual responses for each gambling outcome. Sescousse et al., (2016) was followed to determine the proportions of gambling outcomes, although the present study had fewer trials, resulting in 10 ‘wins’, 10 ‘near-misses before’, 10 ‘near-misses after’ and 30 ‘full-misses’. The ending balance for the session was 750 SEK (≈ 75 €), with each participant receiving the maximum additional reimbursement of 200 SEK (≈ 20 €).

### Data Processing and Analysis

**Fig. 3:**
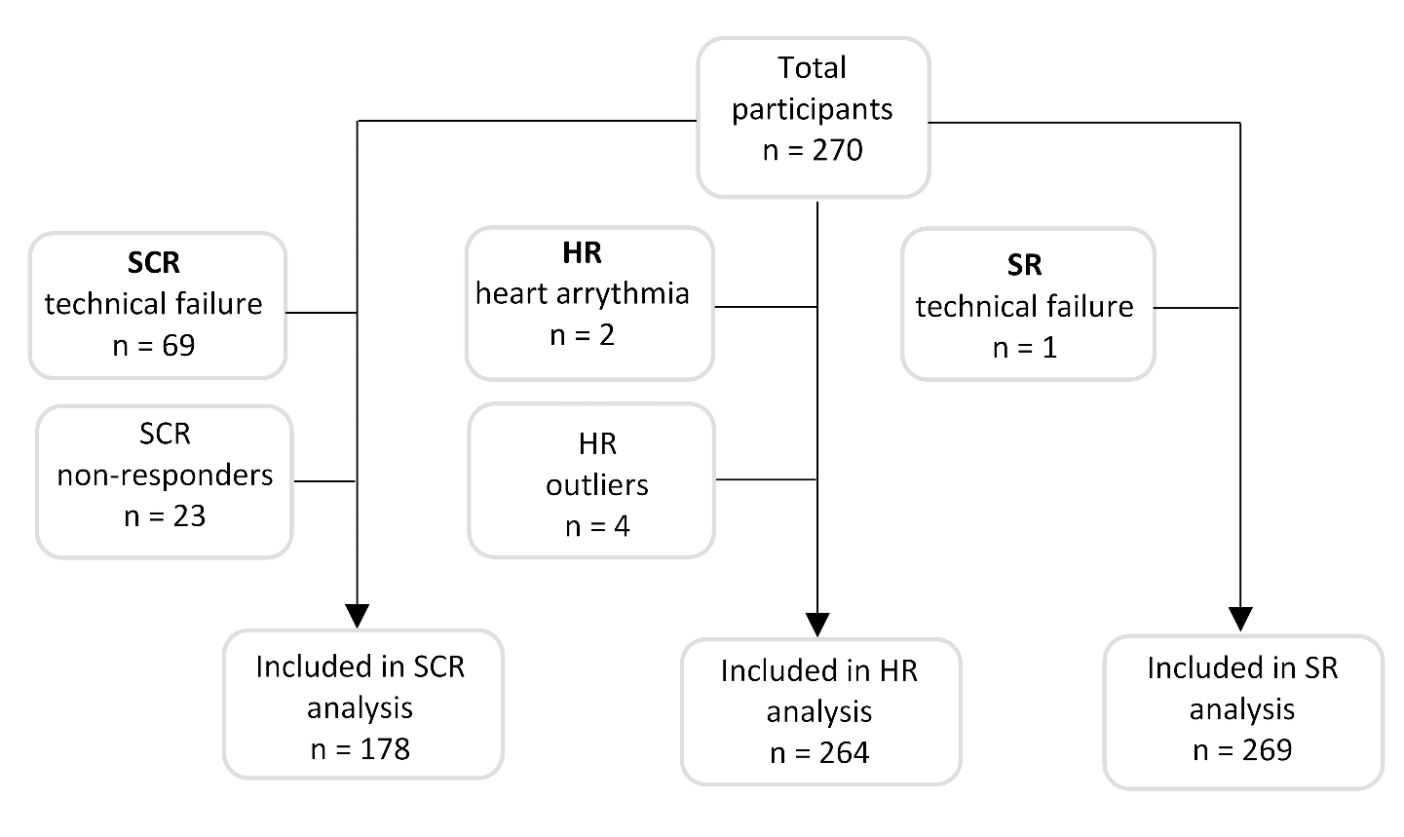
Inclusion procedure for separate analysis of SCR, HR and subjective ratings (SR)

The psychophysiological data were visually inspected prior to analysis to remove recording artifacts attributable to recording noise, excessive movement or electrode detachment (Boucsein, 2012). Artifacts were identified as visually apparent irregularities in the typical EDA and ECG signals (e.g., abnormal short and steep ‘spikes’ in the curve, or longer periods of ‘noisy’ recordings). All of the members of the research group discussed management of the artifacts throughout the visual inspection process. After data management, there was a quality control assessment to exclude any participants with problematic data (Fig. 3). Sixty-nine participants were excluded from the SCR analysis because of a technical failure in the recording equipment. Unfortunately, this excluded all of the participants who scored ‘high’ on PGSI. Given that a proportion of healthy individuals have zero SCRs to emotional stimuli (Venables & Mitchell, 1996), participants were excluded if they had zero SCRs to ≥50% of the winning outcomes. Using this threshold, 23 participants were identified as non-responders and excluded, leaving a total of 178 participants in the SCR analysis. In the HR analysis, two participants were excluded because of heart arrythmia, and four were identified as outliers based on high bpm compared with the group mean (> 2.5 SD). This left 264 participants in the HR analysis. One participant was excluded from all analyses because of technical failure in all recordings, leaving 269 participants in the SR analysis.

The final step in the processing of the skin conductance data was a low-pass filter to remove any frequencies above 1.0 Hz. The data were then exported for analysis in MATLAB, using the *Ledalab* software package (www.ledalab.de). *Ledalab* was used to perform a continuous decomposition analysis of the EDA signal, which separates skin conductance data into continuous signals of tonic and phasic activity. This type of decomposition is recommended when measurements of event-related sympathetic activity that reflect the original properties of the underlying sudomotor nerve activity are of interest (Benedek & Kaernbach, 2010). An SCR was defined as the maximum SCR amplitude in the 1–4 s post-stimulus onset (gambling outcome) time window, minus the 1 s pre-stimulus onset baseline value. The minimum amplitude criterion for SCRs was set at 0.05 µS (Boucsein et al., 2012). The SCR amplitudes were then logarithmically transformed (ln(SCR.amp + 1)) to normalize the data, and summary measures for each gambling outcome were calculated based on the maximum SCR post-stimulus onset amplitudes across all trials.

HR (in beats per minute, bpm) was calculated for every half second, 0–8 s post-stimulus onset. HR summary measures for each gambling outcome were calculated based on Hodes, Cook III, and Lang (1985): initial baseline value (1 s pre-stimulus average HR), initial deceleration component (the minimum 0–3 s post-stimulus value, minus the baseline value) and a subsequent acceleration component (the maximum 2–6 s post-stimulus value, minus the deceleration component).

### Statistical Analyses

All analyses were performed using IBM SPSS Statistics (v26). A repeated-measures analysis of variance (ANOVA) was used to analyse summary measures for the SRs and physiological responses, with significance threshold set at p < 0.05, and Huynh–Feldt and Greenhouse–Geisser corrections used. Post hoc tests included Bonferroni corrections. Three sets of analyses were performed. The first set of analyses had three within-subject factor levels to explore differential responses to the gambling outcomes (wins, near-misses and full-misses) for SCR, ECG and SRs. The second set of analyses focused on the different subtypes of near-misses, using three within-subject factor levels (near-misses before payline, near-misses after payline and full-misses) for SCR, ECG and SRs.

Finally, analysis investigated sex differences in the responses to gambling outcomes (wins, near-misses and full-misses) in two steps. Initially, a repeated measures ANOVA model with three within-subject factor levels (wins, near-misses and full-misses) split by sex was used to investigate responses to each gambling outcome among females and males separately. Secondly, separate two-way ANOVA models for each gambling outcome were performed to explore differences between male and female responses to different gambling outcomes. Of the 269 participants, 35 fell into one of the at-risk gambling categories, ranging from low-risk gambler to problem gambler; 20 participants were considered to be at low risk (2 females, 18 males), 14 at moderate risk (3 females, 11 males) and 1 male was considered to be a problem gambler. Because of zero inflation and large violations of the assumptions for ANOVA caused by the small number of at-risk gamblers, there was no adjustment for problem gambling in the analysis. Cohen (2013) guidelines were followed to assess the magnitude of the effect (η_p_^2^) in the ANOVA models: small = 0.01, medium = 0.06 and large = 0.15. A sensitivity power analysis for sex differences was performed using G*Power software (Faul et al., 2007). With an alpha = 0.05 and power = 0.80, the group sample sizes of N = 140/130 allowed a minimum detectable effect (MDE) of d = 0.30, or η_p_^2^ ≈ 0.02 (Cohen, 2013). Because of smaller group sample sizes of N = 94/84, the SCR analysis allowed an MDE of d = 0.37, or η_p_^2^ ≈ 0.03.

## Results

### Effect of Gambling Outcome

The first analysis was a repeated measures ANOVA model with three within-subject factor levels (wins, near-misses and full-misses). The analysis revealed a significant effect of gambling outcome on SCR amplitudes (*F*(1.44, 254.46) = 164.35, *p* < 0.001, η_p_^2^ = 0.48). The post hoc test with Bonferroni correction revealed greater SCRs for wins compared with both near-misses (*p* < 0.001) and full-misses (*p* < 0.001), and near-misses elicited greater SCRs than full-misses (*p* < 0.001) (Table 1).

**Table 1:** Subjective ratings and psychophysiological responses on the slot machine task [mean (SD)]

	Wins	Near-misses	Full-misses	Post hocs	Effect size (η_p_^2^)
*Subjective ratings*					
Pleased with result	8.23 (1.98)	1.58 (2.14)	1.58 (2.11)	Win > NM **≈** FM	0.83
Continue to play	4.82 (3.05)	4.13 (2.92)	4.11 (2.88)	Win > NM **≈** FM	0.20
Chance of winning	3.14 (2.41)	2.75 (2.23)	2.70 (2.17)	Win > NM **≈** FM	0.10
*Psychophysiology*					
SCR amp (µS)	0.60 (0.30)	0.37 (0.18)	0.32 (0.16)	Win > NM > FM	0.48
HR acceleration	11.46 (4.31)	12.55 (4.37)	11.75 (3.82)	Win < NM > FM	0.10
HR deceleration	4.48 (2.38)	4.03 (2.03)	3.72 (1.75)	Win > NM > FM	0.07

*SCR amp (µS)* = skin conductance response (SCR, max amplitude in microsiemens). *HR* = heart rate (beats per minute). *Subjective ratings* = scale from 1 to 10. *Degrees of freedom*: Subjective ratings, df = 2; EDA, df = 2; HR, df = 2. *Post hoc threshold* = p < 0.05. *Effect size* = partial eta squared (η_p_^2^)

All of the gambling outcomes were followed by a biphasic cardio-vascular response, with an initial deceleration phase (0–2 s post-outcome), followed by a subsequent acceleration phase (2–6 s post-outcome). The effect of gambling outcome was significant for HR acceleration (*F*(1.71, 449.33) = 29.08, *p* < 0.001, η_p_^2^ = 0.10) and HR deceleration (*F*(1.81, 475.73) = 19.44, *p* < 0.001, η_p_^2^ = 0.07), but with different results in the post hoc pairwise comparison tests. Near-misses elicited the largest HR acceleration compared with both wins (*p* < 0.001) and full-misses (*p* < 0.001), but HR acceleration did not differ between wins and full-misses. The largest HR deceleration was elicited by wins, and the deceleration for near-misses was greater than that for full-misses (*p* < 0.001).

There was a significant effect of gambling outcome on the SRs for ‘pleased with result’ (*F*(1.02, 272.65) = 1349.39, *p* < 0.001, η_p_^2^ = 0.83), ‘continue to play’ (*F*(1.15, 308.24) = 66.97, *p* < 0.001, η_p_^2^ = 0.20) and ‘chance of winning’ (*F*(1.30, 349.67) = 32.33, *p <* 0.001, η_p_^2^ = 0.10). The post hoc test with Bonferroni correction revealed that ratings for perceived chance of winning, pleasure and motivation to play were greatest following wins (all *p* < 0.001), with no reliable difference between near-misses and full-misses (Table 1).

### Effects of Different Subtypes of Near-misses

**Fig. 4:**
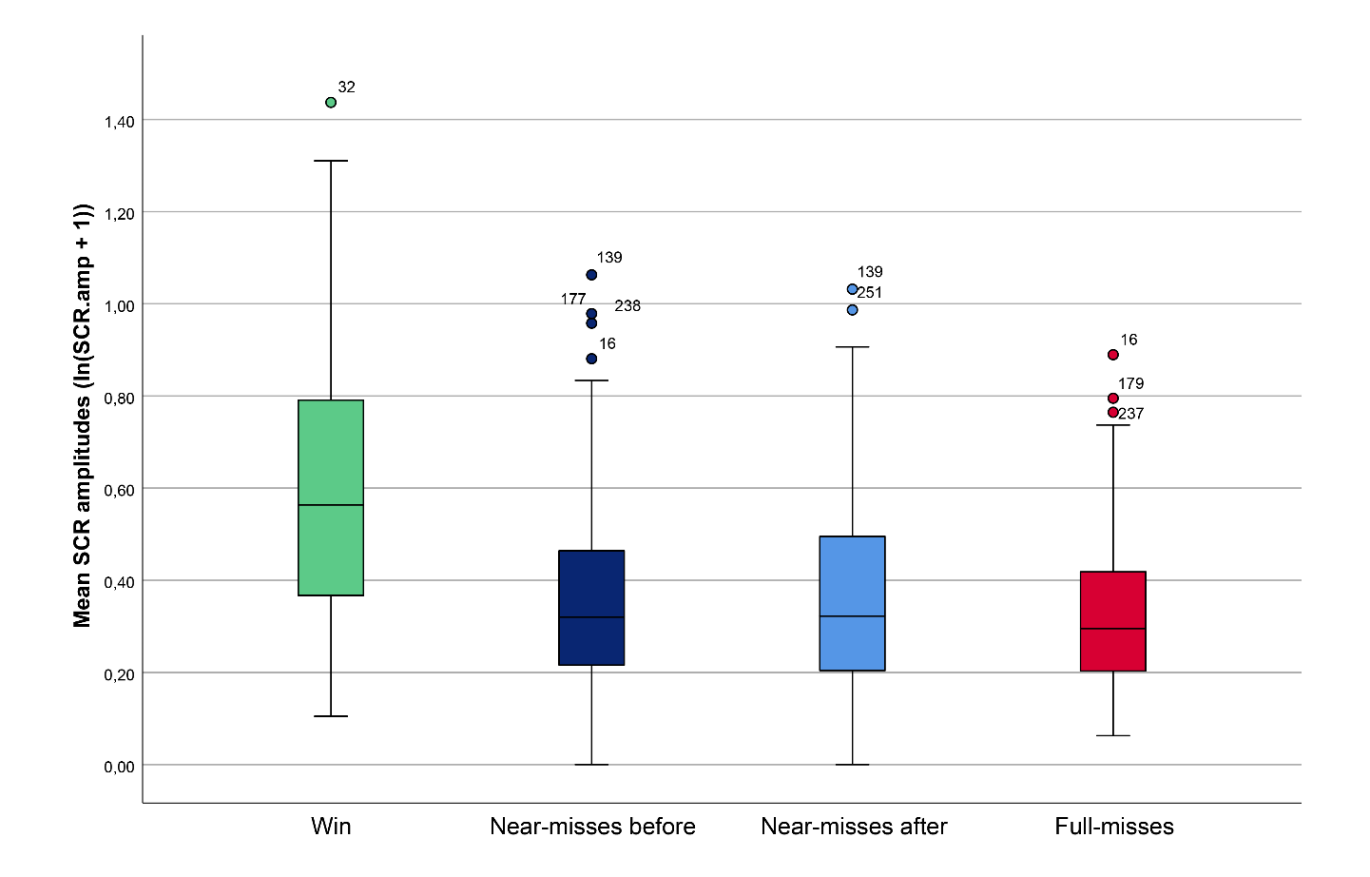
Mean SCR amplitudes (ln(SCR.amp + 1)) following presentation of the different gambling outcomes; wins, near-misses before, near-misses after and full-misses. *Error bars*: 95% CI

The effects of near-misses before the payline and near-misses after the payline were investigated with a three within-subject factor ANOVA model (near-misses before, near-misses after and full-misses). Analysis revealed a statistically significant effect of gambling outcome on SCR amplitudes (*F*(1.88, 333.23) = 5.39, *p* = 0.006, η_p_^2^ = 0.03). Post hoc tests using the Bonferroni correction revealed that both near-miss before and after elicited greater SCRs compared with full-misses (*p* = 0.005, *p* = 0.014, respectively), with no significant difference in SCRs between the two near-miss subtypes (*p* = 1.00) (Fig. 4).

**Fig. 5:**
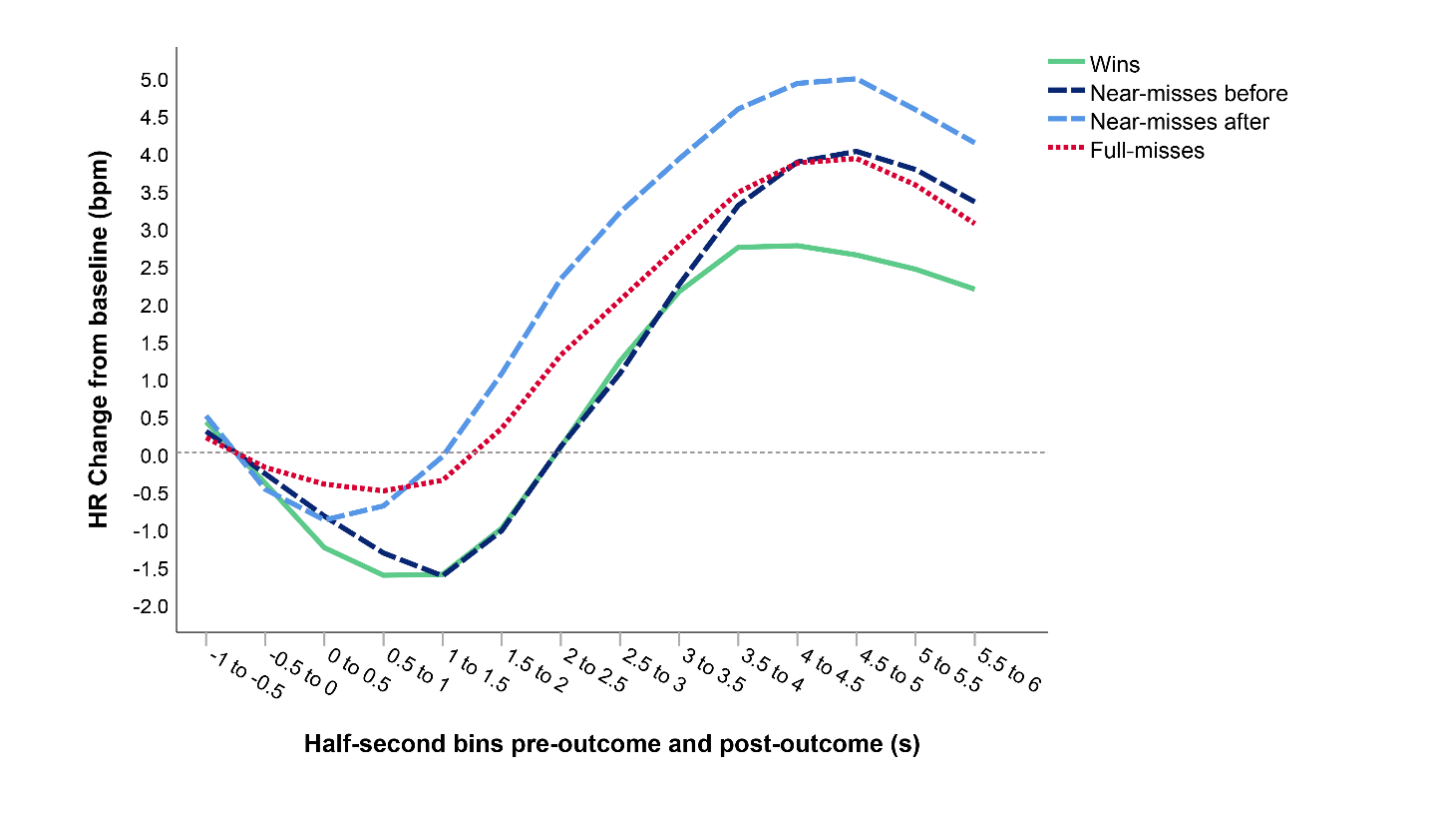
Time-course data displaying changes in heart rate (HR) (from 1 s pre-outcome to 6 s post-outcome), following presentation of the different gambling outcomes; wins, near-misses before, near-misses after and full-misses. The reference line indicates baseline responding, calculated as mean bpm − 1 to 0 s (y-axis). Mean bpm is calculated for every half-second bin, with stimulus outcomes occurring at 0 (x-axis)

The model for HR activity indicated a significant effect of gambling outcome on HR acceleration (*F*(1.91, 502.48) = 22.76, *p* < 0.001, η_p_^2^ = 0.08) and HR deceleration (*F*(1.92, 504.98) = 24.59, *p* < 0.001, η_p_^2^ = 0.09). The results revealed different HR acceleration responses for the near-miss subtypes. Near-misses after elicited greater HR acceleration than both near-misses before (*p* = 0.040) and full-misses (*p* < 0.001). The near-miss subtypes also differed in terms of HR deceleration, with near-misses before eliciting larger HR deceleration than near-misses after (*p* < 0.001). There were no differences in HR deceleration between near-misses after and full-misses (*p* = 1.00) (Table 2). The most prominent divergence between the different gambling outcomes is observed in the acceleration phase, which is consistent with results reported by Clark et al., (2012). The time course of HR changes from 1 s pre-outcome to 6 s post-outcome for all gambling outcomes is presented in Fig. 5. Note that the deceleration phase appears to start prior to the onset of the outcome (Fig. 5).

**Table 2:** Subjective ratings and psychophysiological responses on the slot machine task [mean (SD)]

	Near-misses before	Near-misses after	Full-misses	Post hocs	Effect size (η_p_^2^)
*Subjective ratings*					
Pleased with result	1.62 (2.14)	1.55 (2.17)	1.58 (2.11)	NMB > NMA **≈** FM	0.014
Continue to play	4.36 (2.99)	3.90 (2.92)	4.11 (2.88)	NMB > NMA < FM	0.161
Chance of winning	2.83 (2.29)	2.67 (2.24)	2.70 (2.18)	NMB > NMA **≈** FM	0.029
*Psychophysiology*					
SCR amp (µS)	0.37 (0.21)	0.36 (0.20)	0.32 (0.16)	NMB **≈** NMA > FM	0.030
HR acceleration	12.34 (4.55)	12.76 (4.61)	11.75 (3.82)	NMB < NMA > FM	0.080
HR deceleration	4.44 (2.33)	3.61 (2.34)	3.72 (1.75)	NMB > NMA **≈** FM	0.086

*SCR amp (µS)* = skin conductance response (SCR, max amplitude in microsiemens). *HR* = heart rate (beats per minute). *Subjective ratings* = scale from 1 to 10. *Degrees of freedom*: Subjective ratings, df = 2; EDA, df = 2; HR, df = 2. *Post hoc threshold* = p < 0.05. *Effect size* = partial eta squared (η_p_^2^)

The SR ANOVA model indicated a significant effect of gambling outcome on ‘pleased with result’ (*F*(1.86, 499.08) = 3.87, *p* = 0.024, η_p_^2^ = 0.014), ‘continue to play’ (*F*(1.45, 388.81) = 51.56, *p* < 0.001, η_p_^2^ = 0.16) and ‘chance of winning’ (*F*(1.70, 454.40) = 8.09, *p* = 0.001, η_p_^2^ = 0.03). Although the previous ANOVA model did not reveal a difference between near-misses and full-misses, distinguishing between near-miss subtypes revealed higher perceived chance of winning and motivation to play following near-misses before compared with near-misses after (*p* = 0.002, *p* < 0.001, respectively) and full-misses (*p* = 0.021, *p* < 0.001, respectively). Specifically, near-misses after resulted in lower ratings of ‘continue to play’ compared with both full-misses (*p* < 0.001) and near-misses before (*p* < 0.001). Near-misses before resulted in slightly higher ratings of ‘pleased with result’ than near-misses after (*p* = 0.040). No differences were found in the pleasure ratings between near-misses before and full-misses (*p* = 0.219), nor between near-misses after and full-misses (*p* = 0.679) (Table 2).

### Sex Differences

The final analyses examined sex differences in the physiological responses and SRs in two steps. Initially, repeated measures ANOVA models split by sex were performed. Results revealed a significant effect of gambling outcome on SCR amplitudes for both females (*F*(1.52, 141.32) = 105.04, *p* < 0.001, η_p_^2^ = 0.53), and males (*F*(1.34, 110.88) = 64.26, *p* < 0.001, η_p_^2^ = 0.44). The post hoc test with Bonferroni correction revealed that both females and males had greater SCRs for wins compared with both near-misses (*p* < 0.001, *p* < 0.001, respectively) and full-misses (*p* < 0.001, *p* < 0.001, respectively). Near-misses elicited greater SCRs than full-misses among females (*p* < 0.001) but not among males (*p* = 0.813) (Table 3).

**Table 3:** Sex differences in physiological and subjective ratings (ANOVA)

	N		Mean (SD)		ANOVA	Effect size (η_p_^2^)
	Females	Males	Females	Males		
**SCR amp (µS)**	94	84				
Wins			0.64 (0.31)	0.55 (0.28)	*F* = 4.315, *p* = 0.039	0.024
Near-misses			0.39 (0.19)	0.35 (0.16)	*F* = 2.289, *p* = 0.132	0.013
Full-misses			0.32 (0.16)	0.33 (0.16)	*F* = 0.356, *p* = 0.552	0.002
Post hocs*			Win > NM > FM	Win > NM **≈** FM		
**HR acceleration**	136	128				
Wins			11.48 (4.07)	11.44 (4.57)	*F* = 0.006, *p* = 0.939	0.000
Near-misses			12.27 (4.25)	12.85 (4.50)	*F* = 1.145, *p* = 0.286	0.004
Full-misses			11.66 (3.85)	11.85 (3.79)	*F* = 0.171, *p* = 0.679	0.001
Post hocs*			Win < NM > FM	Win < NM > FM		
**HR deceleration**	136	128				
Wins			4.23 (1.83)	4.75 (2.83)	*F* = 3.171, *p* = 0.076	0.012
Near-misses			4.06 (1.85)	4.00 (2.21)	*F* = 0.055, *p* = 0.815	0.000
Full-misses			3.84 (1.72)	3.59 (1.78)	*F* = 1.311, *p* = 0.253	0.005
Post hocs*			Win **≈** NM **≈** FM	Win > NM > FM		
**Pleased with result**	139	130				
Wins			8.41 (1.95)	8.04 (2.01)	*F* = 2.455, *p* = 0.118	0.009
Near-misses			1.41 (1.88)	1.77 (2.38)	*F* = 1.902, *p* = 0.169	0.007
Full-misses			1.37 (1.86)	1.80 (2.34)	*F* = 2.718, *p* = 0.100	0.010
Post hocs*			Win > NM **≈** FM	Win > NM **≈** FM		
**Continue to play**	139	130				
Wins			5.23 (3.03)	4.38 (3.02)	*F* = 5.291, *p* = 0.022	0.019
Near-misses			4.34 (2.85)	3.91 (2.99)	*F* = 1.471, *p* = 0.226	0.005
Full-misses			4.29 (2.81)	3.91 (2.96)	*F* = 1.169, *p* = 0.280	0.004
Post hocs*			Win > NM **≈** FM	Win > NM **≈** FM		
**Chance of winning**	139	130				
Wins			3.25 (2.24)	3.03 (2.59)	*F* = 0.526, *p* = 0.469	0.002
Near-misses			2.62 (2.02)	2.88 (2.45)	*F* = 0.906, *p* = 0.342	0.003
Full-misses			2.64 (2.01)	2.78 (2.35)	*F* = 0.271, *p* = 0.603	0.001
Post hocs*			Win > NM **≈** FM	Win **≈** NM **≈** FM		

*SCR amp (µS)* = skin conductance response (SCR, max amplitude in microsiemens). *HR* = heart rate (beats per minute). *Subjective ratings* = scale from 1 to 10. *Degrees of freedom*: Subjective ratings, df = 2; EDA, df = 2; HR, df = 2. *Effect size* = partial eta squared (η_p_^2^)

Post hocs* = Differences in responses to each outcome among females and males separately. *Post hoc threshold* = p < 0.05

The effect of gambling outcome was significant for HR acceleration (females: *F*(1.78, 240.60) = 11.32, *p* < 0.001, η_p_^2^ = 0.08, and males: *F*(1.66, 211.06) = 17.93, *p* < 0.001, η_p_^2^ = 0.12) and for HR deceleration (females: *F*(1.86, 251.06) = 3.24, *p* = 0.044, η_p_^2^ = 0.02, and males: *F*(1.80, 228.66) = 18.89, *p* < 0.001, η_p_^2^ = 0.13). For both females and males near-misses elicited the largest HR acceleration compared with wins (*p* < 0.001, *p* < 0.001, respectively) and full-misses (*p* < 0.001, *p* < 0.001, respectively), but HR acceleration did not differ between wins and full-misses (*p* = 1.000, *p* = 0.309, respectively). For males, the largest HR deceleration was elicited by wins, and the deceleration for near-misses was greater than that for full-misses (*p* = 0.028). Females showed no differences in HR deceleration in response to any of the different outcomes (Table 3).

For both females and males there was a significant effect of gambling outcome on the SRs for ‘pleased with result’ (females: *F*(1.01, 139.75) = 858.24, *p* < 0.001, η_p_^2^ = 0.86, and males: *F*(1.02, 131.81) = 537.10, *p* < 0.001, η_p_^2^ = 0.81), ‘continue to play’ (females: *F*(1.18, 162.47) = 51.18, *p* < 0.001, η_p_^2^ = 0.27, and males: *F*(1.12, 143.98) = 18.54, *p* < 0.001, η_p_^2^ = 0.13) and ‘chance of winning’ (females: *F*(1.23, 169.72) = 43.10, *p <* 0.001, η_p_^2^ = 0.24, and males: *F*(1.38, 177.94) = 4.01, *p* = 0.034, η_p_^2^ = 0.03). For both females and males, wins elicited the highest ratings on ‘pleased with results’, but there were no differences in the pleasure ratings between near-misses and full-misses (*p* = 0.339, *p* = 1.000, respectively). For both females and males, wins elicited the highest ratings on ‘continue to play’, but there were no differences in the ratings between near-misses and full-misses (*p* = 0.743, *p* = 1.000, respectively). Among females, wins elicited the highest ratings for ‘chance of winning’, but there were no differences between near-misses and full-misses (*p* = 1.000). Males showed no differences in the ratings for ‘chance of winning’ between near-misses and full-misses (*p* = 0.130) but had higher ratings for wins compared to full-misses (*p* = 0.046) (Table 3).

Secondly, separate two-way ANOVA models for each gambling outcome were performed. Results revealed that females had slightly larger SCRs than males following wins (F(1,176) = 4.315, *p* = 0.039, η_p_^2^ = 0.024). SCRs following near-misses and full-misses did not differ between males and females. There were no sex differences in HR acceleration or deceleration following any of the gambling outcomes (Table 3).

Analysis of the SRs revealed that females scored slightly higher than males on ‘continue to play’ following wins (F(1,267) = *F* = 5.291, *p* = 0.022, η_p_^2^ 0.019). Ratings for ‘continue to play’ following near-misses and full-misses did not differ between males and females. No sex differences were observed in the ratings for ‘pleased with result’ or ‘chance of winning’ following any of the gambling outcomes (Table 3).

## Discussion

### Psychophysiological Responses to Near- and Full-misses

This study used a highly powered design to explore the psychophysiological responses elicited by wins, near-misses and full-misses in a computerized slot machine task. Overall, the results are consistent with previous research, but with a few departures.

As expected, monetary wins produced the largest SCRs, HR deceleration and SRs of perceived chance of winning, pleased with result and motivation to continue to play. SCRs are considered to be reliable indicators of autonomic arousal (Boucsein, 2012), and the current data confirm the arousing effect of gambling wins. This is consistent with previous research showing both increased tonic levels (Coventry & Constable, 1999; Coventry & Hudson, 2001; Diskin & Hodgins, 2003; Moodie & Finnigan, 2005; Sharpe, 2004; Wilkes et al., 2010; Wulfert et al., 2005, 2008) and phasic responses (Clark et al., 2012, 2013; Dixon et al., 2010; Lole et al., 2012, 2014). Increased arousal patterns for wins accompanied by the highest ratings for motivation, pleasure and expectancy of winning are consistent with theories suggesting that physiological arousal reinforces gambling (Blaszczynski & Nower, 2002; Brown, 1986; Sharpe, 2002; Sharpe & Tarrier, 1993). Large autonomic responses to gambling wins are to be expected, not only given the excitement of winning money, but also given the visual and auditory sensory feedback accompanying this outcome. In this study, winning was accompanied by a large HR deceleration response, which is believed to reflect increased sensory processing and attention orienting (Bradley et al., 2001; Codispoti et al., 2001).

Importantly, the response patterns observed in the current study revealed differential autonomic responses between near-misses and full-misses. Despite having the same monetary outcome as full-misses, near-misses elicited significantly larger skin conductance and cardio-vascular responses, although mean differences in SCRs between near-misses and full-misses were relatively small. Nevertheless, greater SCRs for near-misses than for full-misses are consistent with findings reported by Clark et al., (2012; 2013), as is the greater HR acceleration for near-misses relative to both wins and full-misses. Some authors infer that increased SCRs to near-misses may be associated with a sense of frustration and negative affect (Clark et al., 2012, 2013; Dixon et al., 2011). Differences in HR deceleration were also observed for the gambling outcomes, with larger deceleration following wins and near-misses, suggesting enhanced orienting for these outcomes. Interestingly, Fig. 5 shows that the HR orienting response starts prior to the reel coming to a complete standstill. This may be caused by the anticipation that builds during the gradual slowing of the reel before the outcome is unambiguously revealed. However, although Clark et al., (2012; 2013) used a slot machine task with the same anticipation interval, they did not observe this difference in HR deceleration.

Drawing upon the design by Clark et al., (2012; 2013), this study used trial-by-trial ratings to capture subjective reports of pleasure, motivation to play and perceived chance of winning. Initial analyses revealed that, relative to full-misses, near-misses did not generate differential SRs of pleasure and motivation to play, that have been reported by others (Clark et al., 2009, 2012; Qi et al., 2011). The perceived chance of winning did not differ between gambling outcomes, which is consistent with previous reports (Billieux et al., 2012; Clark et al., 2013). However, it should be noted that the timing of SRs used in this study differed slightly from that used in previous studies in which participants rated the ‘chance of winning’ after the icon choice, and then they rated ‘pleased with result’ and ‘continue to play’ following the outcome. In the present study, participants gave all three ratings following the outcome, which may have reduced the sensitivity of the ratings. It is also important to consider the repetitive nature of this manipulation, which could create a sense of boredom that diminishes the ability of the ratings to capture reliable subjective perceptions generated by gambling outcomes.

Interestingly however, differences in the SRs along with cardio-vascular responses emerged when comparisons were made between the two near-miss subtypes. Near-misses after the payline were characterized by large HR acceleration and low reports of motivation to play (even lower than for full-misses), whereas near-misses before the payline were associated with greater HR deceleration and higher subjective states of motivation (continue to play) than both near-misses after and full-misses, as well as slightly higher affective responses (pleased with results) than near-misses after. These motivational and affective responses are consistent with previous results indicating aversive responses for near-misses after, including findings reported by Clark et al., (2013), Sharman et al., (2015) and Wu et al., (2017). The current study further extends previous findings by revealing a higher perceived chance of winning following near-misses before compared with both near-misses after and full-misses, along with the HR responses previously described, which Clark et al., (2013) did not observe. However, although HR responses, the perceived chance of winning, pleasure and motivation all differed according to the near-miss position, this was not the case for SCRs. Clark et al., (2013) observed increased SCRs for near-misses after. Both near-miss subtypes combined generated distinct SCRs in the previous analysis, but the present study did not find evidence for a difference between the two near-miss subtypes in the elicited SCRs. However, it is noteworthy that a large proportion of participants were excluded from the SCR analysis in this study because of a technical failure, which may have reduced statistical power in these analyses. Nevertheless, the remaining proportion of participants was still larger than that included in Clark et al., (2013).

Although near-misses in general generated distinct psychophysiological responses, the data suggest that the large HR acceleration paired with low pleasure and motivational ratings indicate frustrating effects that are primarily driven by near-misses that occur after the payline. In contrast, based on higher subjective states of motivation to play, pleasure and perceived chance of winning, near-misses before appeared to be more invigorating. Further, there were no differences in the subjective reports for near-misses in general relative to full-misses, but differences emerged between the near-miss subtypes. Hence, the differential physiological, affective and motivational effects observed are consistent with the theory that subtypes of near-misses constitute two distinct upward counterfactuals (additive and subtractive) affecting emotion and motivation (for a review, see Clark et al., (2013)).

### Sex Differences

One novel aim of the current study was to explore potential sex differences in psychophysiological responding to gambling outcomes in a computerized slot machine task. Initially, female, and male responses to wins, near-misses and full-misses were analyzed separately. In terms of the physiological measures, both females and males showed the largest HR acceleration to near-misses compared with all other outcomes. However, females also showed greater SCR responses for near-misses compared to full-misses, while males did not. This suggests that females were responsible for the larger SCR responses to near-misses compared to full-misses observed in the overall sample. Males showed different patterns of HR deceleration depending on the outcome, with the largest HR deceleration elicited by wins, and greater responses to near-misses than full-misses. Females, however, showed similar HR deceleration responses to all outcomes. This indicates sex differences in orienting responses, and that males were responsible for the outcome related variation in HR deceleration observed in the overall sample. In addition, females and males displayed similar patterns of pleasure and motivation to play following all outcomes. Females differed in terms of their perceived chance of winning, which was higher following wins compared to both near-misses and full-misses. Males showed similar levels of perceived chance of winning following all outcomes, but with slightly higher ratings following wins compared to full-misses, which barely reached significance.

Secondly, separate analyses for each gambling outcome were performed to explore differences between male, and female responses to different gambling outcomes. Although previous analysis indicated that females and males displayed different contributions to the variations in autonomic responses observed in the overall sample (SCR responses among females, and HR deceleration responses among males), these differences were not evident in the subsequent analysis comparing female and male responses. Sex differences were only visible through larger SCRs and higher scores on ‘continue to play’ following wins among females (medium effect sizes), suggesting increased reactivity and motivation following monetary wins. Although this suggests sex differences in the emotional processing of monetary gambling rewards, the interpretation of these results is not entirely clear. The increased motivation and SCRs for females following wins are difficult to reconcile with research showing enhanced arousal among males during gambling (Franco et al., 2009), or neural research suggesting that men have greater emotional sensitivity to reward (Dhingra et al., 2021; Garrido-Chaves et al., 2021; Grose-Fifer et al., 2014; Santesso et al., 2011). However, these studies used tasks with greater risk than the slot machine task that was used in the current study. Thus, the results challenge the view that males are generally more reward-sensitive than females and suggest that sex differences in reward processing depend upon the level of risk associated with the game.

Research suggests that negative mood states (depression, loneliness, boredom, anxiety) may increase the risk for problematic gambling behaviour, especially in women (Grant et al., 2012; Sundqvist & Rosendahl, 2019; Tschibelu & Elman, 2010), who also appear to show an accelerated course of problem gambling (Martins et al., 2002; Zakiniaeiz et al., 2017). Therefore, because arousal is a significant reinforcer of problem gambling, the arousal patterns of women playing slot machine games are of great concern, especially as slot machine gambling is more prevalent among women gamblers (Grant et al., 2012; Håkansson & Widinghoff, 2020; Potenza et al., 2006; Stark et al., 2012; Tavares et al., 2003).

However, it should be noted that SCR is a complex measure that is sensitive to environmental conditions such as season and time of day, and females have been shown to be more responsive than males to these conditions (Venables & Mitchell, 1996). Despite efforts to regulate temperature and humidity, it is possible that varying environmental conditions influenced the results of this study. Hormone levels and menstrual cycle also mediate electrodermal activity and SCR (Boucsein, 2012; Gómez-Amor et al., 1990), but they were not controlled for in this study.

### Strengths and Limitations

The major strength of this study is the inclusion of a large sample of young adults, recruited from a community-based cohort, resulting in a sufficient subset of males and females to detect a minimal effect of d = 0.3 (η_p_^2^ ≈ 0.02). Given the increasing need to improve reproducibility, the current study is an important contribution to the research field of psychophysiology in gambling. Although the computerized slot machine task was simplified compared with real life gambling, the procedures used were still able to capture differential phasic responses to specific gambling stimuli, thereby strengthening the validity of a laboratory gambling setting.

The limitations of the study mainly concern context and setting. As part of a larger experimental session in which the participants performed several other tasks before the slot machine task, the potential effects of boredom and disinterest cannot be ruled out. Second, the subjective trial-by-trial ratings must be interpreted with caution, as these are not direct behavioural measures and, as noted, their reliability may be in doubt. Nevertheless, the small but significant differences in some of the SR analyses provide an indication that differences in higher cognitive processing to near- and full-misses do occur, but this requires further study. Third, no adjustments were made for problem gambling because of the small proportion of participants with any gambling experience. Hence, the results cannot be generalized to problem gamblers. Several other factors such as hormonal levels, medication, psychiatric or neuropsychiatric disorders or personality traits are also possible mediators, but they were not controlled in this study. Finally, a large proportion of participants were excluded from the SCR analysis because of a technical failure, but the included sample was still relatively large.

## Conclusions

To our knowledge, this is the first study to investigate sex differences in psychophysiological responding to wins, near-misses and full-misses in a slot machine gambling task, using a large community sample of young adults. The current study demonstrated that wins, near-misses and full-misses in a slot machine gambling task generate differential psychophysiological responses in young adults. The results indicated that near-misses are complex, multifaceted events that can produce conflicting emotional responses depending upon their characterization (Clark et al., 2013). Near-miss gambling outcomes are structural characteristics believed to be linked to the addictive properties of games of chance that facilitate continued play (Griffiths, 1993; Parke & Griffiths, 2006; Reid, 1986). Such theories view near-misses as uniform events, describing them simply as almost hitting the jackpot, without considering different subtypes of near-misses. However, the data from this study suggest that this distinction is highly relevant to the understanding of near-miss psychology and the effects of such events on gambling behaviour.

The female and male participants in this study responded differently to wins, both physiologically and via self-reports. Gender is known to influence patterns of gambling behaviour, underlying motivations to gamble and the development of problem gambling. Gambling is still more prevalent among men than women. Consequently, most of the experimental research on gambling has used male samples and gendered approaches are still limited. Results from the current study emphasize the importance of considering sex differences in experimental research on autonomic responses to gambling events, the mechanisms of gambling and the role that autonomic arousal plays in problematic gambling behaviour among women and men.

## Data Availability

The data used in the research cannot be publicly shared due to ethical or legal restrictions but are available upon request.
